# The leading region of many plasmids is adapted for translational efficiency

**DOI:** 10.1093/nar/gkag810

**Published:** 2026-08-17

**Authors:** Liam P Shaw, Arancha Peñil-Celis, Manuel Ares-Arroyo, Lijuan Luo, Tatiana Dimitriu, Fernando de la Cruz

**Affiliations:** School of Biochemistry and Biomedical Sciences, University of Bristol, Bristol BS8 1QU, GB; Instituto de Biomedicina y Biotecnología de Cantabria (Consejo Superior de Investigaciones Científicas – Universidad de Cantabria), 39011 Santander, Spain; Institut Pasteur, Université de Paris Cité, CNRS UMR3525, Microbial Evolutionary Genomics, 75724 Paris, France; Institute of Infection, Veterinary and Ecological Sciences, University of Liverpool, Liverpool L69 7ZX, GB; School of Biology, University of St Andrews, St Andrews KY16 9ST, GB; Instituto de Biomedicina y Biotecnología de Cantabria (Consejo Superior de Investigaciones Científicas – Universidad de Cantabria), 39011 Santander, Spain

## Abstract

To successfully transfer, conjugative plasmids must overcome a recipient cell’s defence mechanisms. The leading region of many plasmids—the first DNA to enter—is known to be enriched in anti-defence genes. Here, we investigate whether selective pressure from defence systems has also affected its sequence composition. We consider two possibilities: greater target depletion (to avoid triggering defence systems) or greater codon adaptation (to rapidly express anti-defence genes). We analyse 1751 conjugative plasmids in 13 plasmid taxonomic units in Enterobacterales. First, we test GC content and depletion of short palindromic motifs (4–8 bp), a potential signature of selective pressure from Type II restriction-modification systems. The leading region is GC-rich but not consistently depleted in these motifs. Then, we use the codon adaptation index (CAI) to assess codon usage. Leading regions in plasmids with MOB-F and MOB-P relaxases are more adapted to optimal host codon usage, consistent with higher translational efficiency, with anti-restriction genes showing particular high CAI. We demonstrate the potential of codon adaptation to uncover new plasmid biology by identifying a single-stranded promoter in the F plasmid, consistent with independent experimental findings. Our findings emphasize the intensity of the selective pressure that plasmids face from defence systems.

## Graphical Abstract

**Graphical Abstract:**
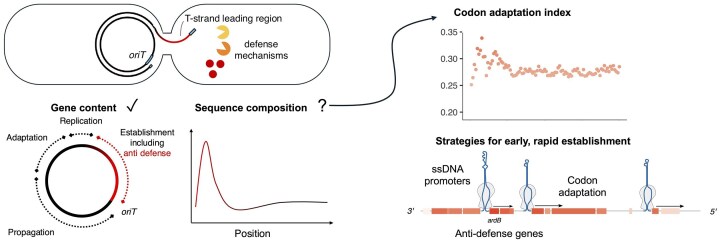

## Introduction

To date, researchers have identified over 250 molecular systems that reduce or prevent infection by mobile genetic elements (MGEs) in bacteria [1]. Whether these so-called ‘defence’ systems are conceptualized as a pan-immune system [2] or alternatively as weapons of inter-MGE conflict [3], any effective system will select for evasion in the MGEs it acts against. Most efforts on understanding evasion have focused on phage and their arms race with bacteria [4–8]. Although it is established that plasmids carry anti-defence genes [9], far less is known about their strategies for evasion. Understanding plasmid evasion is important to understand horizontal evolution, as well as for synthetic biology approaches to engineer MGEs or to block the spread of plasmid-encoded antimicrobial resistance.

During conjugation, a plasmid is transferred from a donor into a recipient as single-stranded DNA (ssDNA) before circularisation and conversion to double-stranded DNA (dsDNA). This is a rapid process, taking just 4 min for the 100 kb F plasmid [10], and always proceeds in the same way relative to the plasmid’s origin of transfer (*oriT*) [11]. The leading region is the first portion of the plasmid to enter the recipient cell and is highly enriched in anti-defence genes [12]. The immediate expression of these genes during transfer, identified in the 1990s and referred to as ‘zygotic induction’ [13], appears to be crucial for plasmid establishment [14].

Selective pressure from defence systems may also shape the leading region’s sequence as well as its gene content. Here, we consider two ways: greater target depletion (to avoid triggering target-based defence systems) or greater codon adaptation (to express anti-defence genes more quickly). To investigate both these possibilities in a dataset of well-characterized hosts, we tested them in 1751 plasmids from within the bacterial order Enterobacterales, including those carrying multiple AMR genes of clinical relevance such as *bla*_NDM-1_, *bla*_CTX-M_, and *bla*_OXA-48_.

For target avoidance, we initially decided to analyse the targets of the most common defence systems, Type II restriction-modification (RM), which are present in around 40% of bacterial genomes and mostly target 4–8 bp palindromic motifs [1]. In a previous study, we showed that plasmids tend to be depleted in Type II RM targets consistent with their known taxonomic range [15], and here we followed that approach to test whether this target depletion is stronger in the leading region. *A priori*, any depletion of RM targets would be surprising since the leading region enters the recipient as ssDNA, and although some Type II restriction enzymes can have activity against secondary structures formed by ssDNA [16], most Type II RM systems target only dsDNA. However, if complementary strand synthesis always proceeds in the same way, then it could be possible for Type II RM selective pressure to be stronger on some regions compared to others based on their temporal conversion to dsDNA.

For codon adaptation, our starting point was that speed matters during plasmid transfer. Previous work on the leading region has emphasized the rapid transcription of its anti-defence genes while still ssDNA [10, 12, 14, 17, 18], but the subsequent step of translation has been overlooked. This is despite these processes having similar rates: assuming maximal efficiency of ∼85 nucleotides per second [19], a 1-kb gene in *Escherichia coli* may take 10 s to be transcribed into mRNA and 10 s to be translated into protein. This may seem short, but it is ∼10% of the conjugative transfer time of the F plasmid, and inefficient translation will further delay it. Translation can be made more efficient through codon adaption: most amino acids can be specified by different codons, and certain codons lead to both faster elongation and greater mRNA stability [20]. These optimal codons can be defined by examining a set of highly expressed chromosomal genes. We used this approach, quantifying codon usage with an established method [21] and comparing the region to the rest of the plasmid.

Our main finding is that the sequence of many leading region shows differences in codon usage compared to the rest of the plasmid, suggesting adaptation for translational efficiency. Leading region genes have a higher codon adaptation index (CAI) and anti-restriction genes in particular have significantly higher scores. Using this improved understanding, we were able to identify a single-stranded promoter in the leading region of the F plasmid. Analysing codon usage may help identify novel features of plasmids, including new anti-defence genes, and help optimize novel conjugative delivery systems.

## Materials and methods

We retrieved 19 017 bacterial plasmids from RefSeq200 and clustered them into related groups of Plasmid Taxonomic Units (PTUs) [22]. We selected the 50 most prevalent PTUs consisting of 4753 plasmids across 37 genera for initial analysis.

### Detection of leading regions

We followed a previously described procedure to identify leading regions [23]. We first annotated all plasmids with prodigal v2.6.3 with the -p meta flag [24] and then used CONJScan v2.0 [25] to detect those with a relaxase, and thus able to be mobilized by conjugation. We discarded 16 PTUs that seemed incapable of moving by conjugation: 11 in which no conjugative trait was found (749 plasmids) and 5 phage-plasmids (279 plasmids). For the remaining 34 PTUs (3725 plasmids) we screened for experimentally validated *oriT* sequences, detecting at least one *oriT* in 2546 plasmids across 20 PTUs. To accurately identify the leading region of the plasmids, we discarded 336 plasmids that contained >1 *oriT* and/or >1 relaxase (except for PTU-X1 and X3, known to have two functional copies of the *oriT*_R6K_) [8], retaining 2210 across 19 PTUs. Although there is no fixed definition for the size of the leading region, it is typically taken to be at least 10 kb e.g. for the F plasmid it is ∼13 kb [. We therefore decided to only analyse plasmids >20 kb, which led to discarding six small mobilizable PTUs from further analysis (median size 4.3 kb, maximum 12.9 kb). We also discarded PTU-HI2 since >50% of the plasmids within it lacked a known *oriT*. In summary, we could confidently identify a leading region in 1751 conjugative plasmids across 13 PTUs, the vast majority of which (1736, 99.1%) were from within Enterobacterales (Supplementary Table S1).

### RM system prevalence

For each of the 37 genera with plasmids in our initial dataset, we downloaded a random sample of at most 100 complete bacterial genomes from RefSeq (3335 genomes) and then used rmsFinder v1.0.0 (https://github.com/liampshaw/rmsFinder) with default thresholds to predict the presence of Type II RM systems. We detected 1335 systems with confidently predicted motifs in 1020 genomes, with 77 unique targeted motifs corresponding to 213 motifs after deambiguation [e.g. CCWGG = (CCTGG, CCAGG)]. The majority of RM motifs were of length *k* = 5 and *k* = 6 (64/76, 83%); within Enterobacterales, there were 89 such motifs across 17 genera, with a median prevalence of 2% per genera. We used this to produce a database of RM systems with known targets across genera (Supplementary Table S2).

### Depletion of RM motifs

Palindromes have previously been used as a proxy for Type II RM targets [6, 7, 15], and we also use this approach here for initial analysis. Because a DNA palindrome of length *k* is determined by each of its three bases matching with its complement at the right position in the last three bases, the expected density of palindromic *k*-mers in a long sequence with a GC content of *p* is given by:


\begin{eqnarray*}
d = {{\left( {\frac{{2{{p}^2} - 2p + 1}}{2}} \right)}^{\frac{k}{2}}}.
\end{eqnarray*}

For example, for a sequence with GC content of 50% we get *d*=(1/4)^3 ^= 0.01565 per base pair, corresponding to a palindrome density of ∼15.6 per kilobase. For a sliding window over a plasmid, we can thus calculate the palindrome depletion along it: the difference between the observed number of palindromic *k*-mers in the window and the theoretical expectation.

We also used RMES v3.1.0 [26] to calculate a more robust representation score for all *k*-mers (sequence motifs of length *k*). RMES (https://forgemia.inra.fr/sophie.schbath/rmes) compares the expected number of occurrences of a *k*-mer to the observed occurrences to calculate a representation score, with negative scores indicating under-representation. We used the maximal model, which uses the (*k-*1)-mer sequence composition to calculate expected occurrences, constituting the most conservative approach because it uses as much information about sequence composition as possible. We compared the leading and lagging regions of plasmids with a fixed length of 10 kb to control for sequence length.

### Gene prediction and functional annotation

After finding protein-coding genes on all plasmids with prodigal v2.6.3 with the -p meta flag [24], we performed functional annotation with eggNOG-mapper (version emapper-2.1.13) [27] based on eggNOG orthology data [28] and diamond for sequence searches [29]. 79.7% of predicted proteins had a hit (163 961 of 205 673) and 63.3% (130 171) were assigned to an informative COG one-letter code (i.e. excluding S, R, and -). We detected AMR with AMRFinderPlus (software v4.2.7, db v2026-03-24.1) with default parameters (>0.5 coverage, >0.9 identity) [30]. We searched for defence and anti-defence systems with DefenseFinder v2.0.1 [1, 9] using defense-finder-models v2.0.2 and CasFinder v3.1.0. This identified 1858 defence genes and 4397 anti-defence genes in 1617 and 3174 systems, respectively (Supplementary Table S3 gives 7004 individual HMM hits with evalue <10^−10^). We searched for tRNAs in all plasmids with trnascan-SE v2.0.12 [31] to confirm that plasmids were dependent on host tRNAs for expression. Only three plasmids had non-pseudo tRNAs reported: NZ_CP017387.1 (PTU-C), NZ_CP030077.1 (PTU-C), and NZ_CP040265.1 (PTU-FE). These are likely the result of previous chromosomal integration and excision.

### Codon usage analysis

The optimal set of codons for an organism are those that occur most frequently in genes translated at high abundance for a given amino acid i.e. for a given amino acid, there is a optimal codon. These optimal codons get a weight *w *= 1, and synonymous codons for the same amino acid get a weight defined by their relative usage frequency to the optimal codon. The CAI of a gene is defined as the geometric mean of the w-values of its *M* codons [21]:


\begin{eqnarray*}
\mathrm{ CAI} = {{\left( {\mathop \prod \limits_{i = 1}^M {{w}_i}} \right)}^{\frac{1}{M}}}.
\end{eqnarray*}

CAI ranges from 0 to 1, with 1 corresponding to optimal codon usage. Since *n* = 892 (50.9%) plasmids were from *Escherichia*, we used the *E. coli* codon weight table calculated by Lucks *et al*. [32] based on 27 very highly expressed genes including the ribosomal genes [33]. For context, the median CAI in the *E. coli* K12 genome is 0.321, and values range from 0.066 (*mgtL*) to 0.830 (*lpp*, from the set of genes used to define optimal codons). We verified that we could recalculate the 61 codon weights from *E. coli* K12 MG1655 matching the figures from Lucks *et al*. [32], then also calculated the analogous weights for other species using blast hits in reference genomes for the 27 highly expressed genes (Supplementary Table S4). Within Enterobacterales we found high correlation with the *E. coli* codon weights: *Salmonella enterica* (GCF_000006945.2) Pearson’s *r* = 0.987, *Klebsiella pneumoniae* MGH78578 (GCF_000016305.1) *r* = 0.986, *Citrobacter freundii* (GCF_904859905.1) *r* = 0.997, and *Enterobacter hormaechei* (GCF_019048245.1) *r* = 0.947. We calculated CAI for all ORFs found with prodigal in plasmids (Supplementary Table S5).

## Results

### Leading regions are associated with anti-defence genes

**Figure 1.**
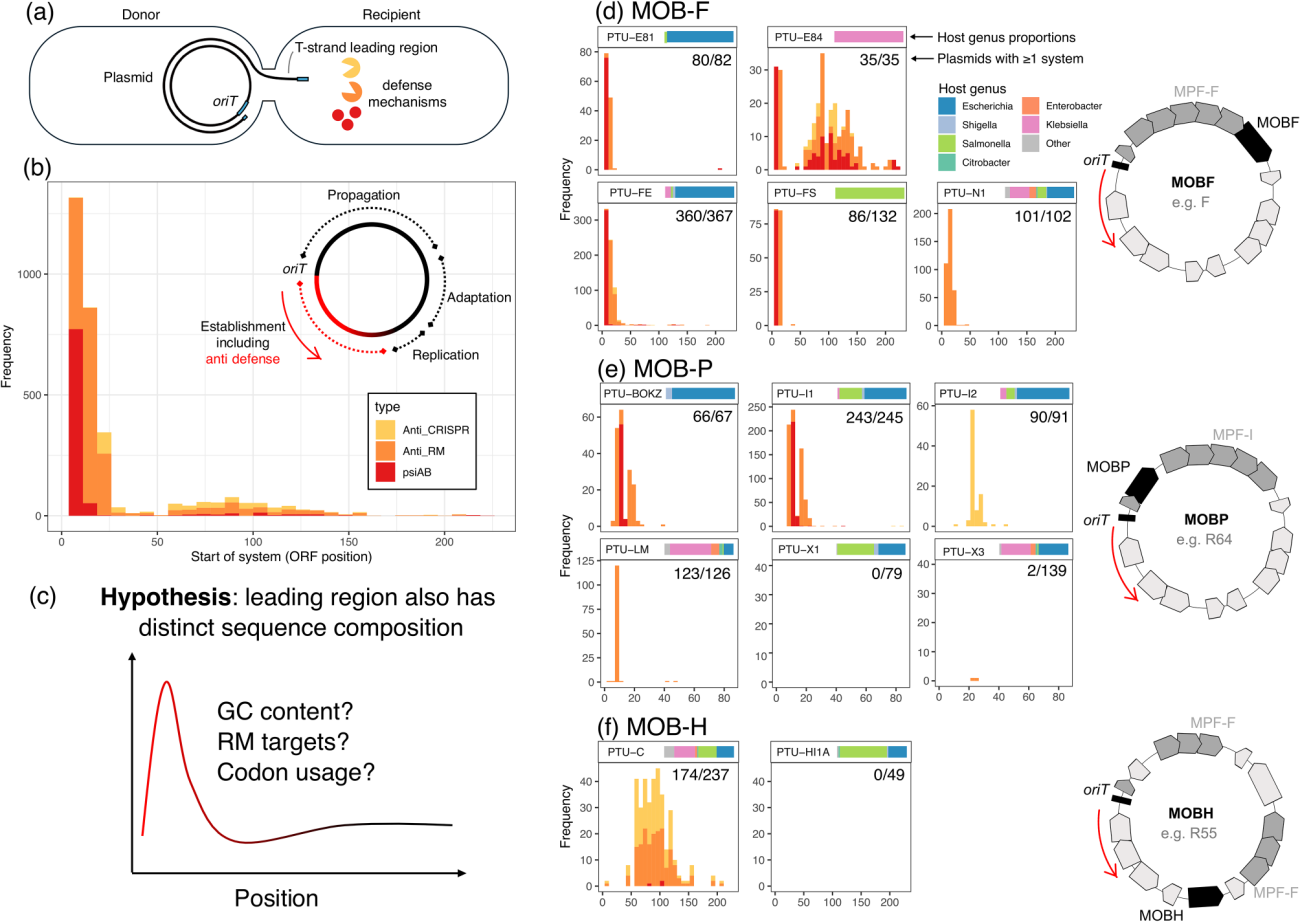
The leading region of many conjugative plasmids is enriched in anti-defence genes. (**a**) Schematic showing conjugative transfer of a plasmid from a donor into a recipient. The leading region on the transfer strand (T-strand) is under strong selective pressure from defence systems including RM, CRISPR-Cas, and abortive infection. (**b**) The leading region is enriched in anti-defence genes, shown here for over 1751 plasmids in 13 PTUs found mainly in Enterobacterales. Inset shows a schematic of plasmid organization following Samuel *et al*. [12]. (**c**) We hypothesize that this selective pressure from defence systems also shapes the leading region’s sequence composition. (**d**–**f**) Enrichment of anti-defence systems in the leading region (frequency i.e. absolute numbers), subsetted by mobility type (the most common relaxase detected within the PTU, with diagrams showing indicative plasmid organization) and then by PTU. *X*-axis shows ORF position as in panel (b). Inset bars in facet titles show host genera proportions, numbers in top-right corner of plots give the number of plasmids with at least one anti-defence system detected. Some PTUs have no anti-defence systems detected (PTU-X1, PTU-HI1A). For individual anti-defence HMM hits (evalue < 10^−10^) see Supplementary Table S3. Not shown in panels (b) and (e) is a single anti-Pycsar system detected in one PTU-BOKZ plasmid (NZ_CP023144.1).

We identified the leading region using an existing method to find the sequence following the *oriT* in the correct orientation i.e. on the T-strand (Fig. 1a, see the “Materials and methods” section). We could confidently locate the leading region in 13 conjugative PTUs within Enterobacterales (1751 plasmids of which 1736 from Enterobacterales, median length 91 kb range 21.9–329.3 kb, median length per PTU 42–217 kb). Conjugative transfer of ssDNA induces defence mechanisms such as the bacterial SOS response [34], but many plasmids can carry anti-defence genes that are expressed early in conjugation. These include *ardA*, encoding an anti-restriction protein that inhibits the endonuclease activity of Type I systems [35], or *psiB*, encoding a protein which inhibits the SOS response [36]. As previously reported, the leading region is enriched in these anti-defence genes [12] and we reproduced this result in our dataset (Fig. 1b), although we note that there are differences by anti-defence mechanism, with anti-CRISPR systems further from the *oriT* than anti-RM systems, which may be because CRISPR takes longer to act once the plasmid enters and/or because there are lower numbers of targets on the plasmid. Our hypothesis was that selective pressure from defence systems could also lead to distinct sequence composition as well as gene content (Fig. 1c). It should be noted that anti-defence enrichment patterns differ by relaxase type (Fig. 1d–f). PTUs where plasmids carried a MOB-F relaxase showed a consistent leading region peak in anti-defence systems, as did MOB-P relaxase PTUs, but MOB-H PTUs showed no detectable peak and some PTUs had no anti-defence systems detected. We return to these observations below.

### Evidence for GC richness and motif depletion

**Figure 2.**
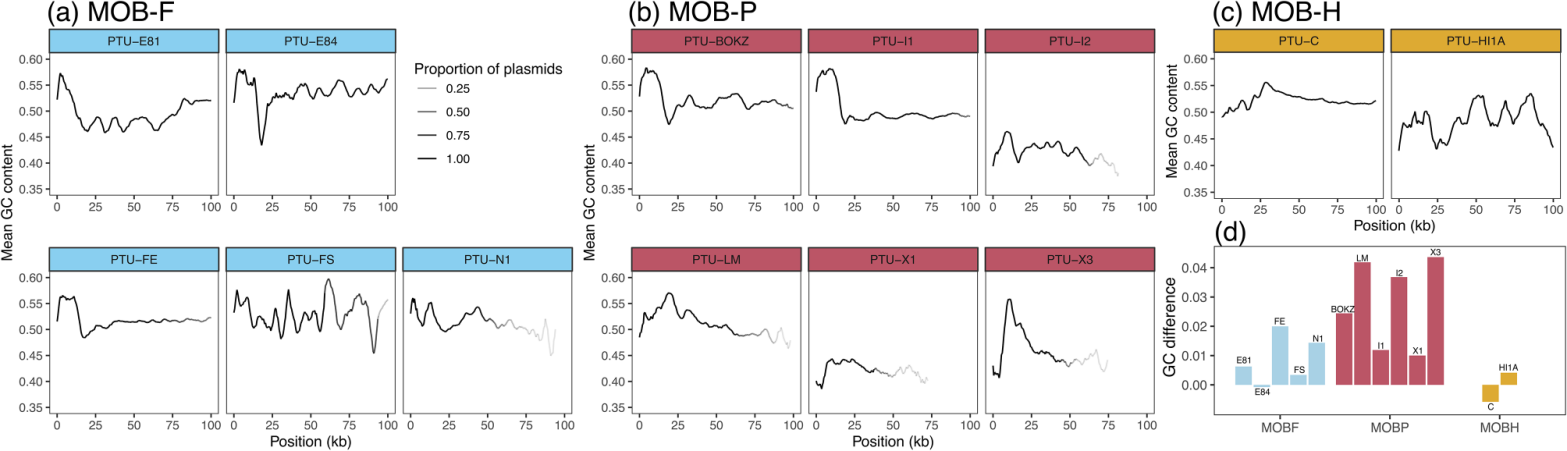
The leading region has higher GC content in many PTUs. Mean GC content in non-overlapping 1-kb windows shown for (**a**) MOB-F (**b**) MOB-P, and (**c**) MOB-H plasmids, faceted by PTU. Plots are truncated at 100 kb with line intensity showing the proportion of plasmids being averaged over within the PTU at each position. (**d**) The difference in mean GC content between the leading 25 kb and the rest of the plasmid for each PTU.

**Figure 3.**
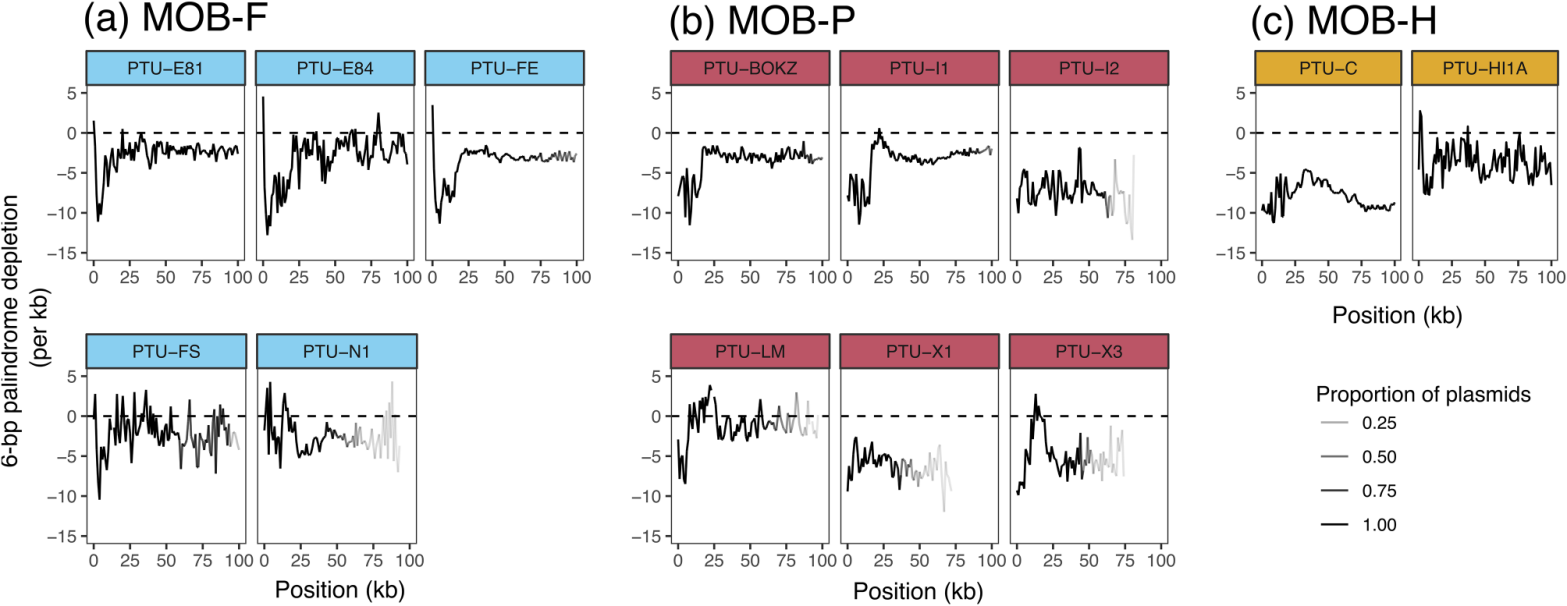
The leading region of some PTUs is depleted in 6-bp palindromes. The difference between observed palindrome density in nonoverlapping 1-kb windows and the theoretical density expected from GC content (see the “Materials and methods” section), such that lower values show depletion below expectation. Line intensity indicates proportion of plasmids being averaged over. PTUs are grouped by MOB relaxase type (**a**) MOB-F (**b**) MOB-P, and (**c**) MOB-H.

The leading region had higher mean GC content in many plasmids, with differences between PTUs (Fig. 2a–c). Ten out of 11 PTUs associated with MOB-F and MOB-P relaxases had higher GC in the leading 25 kb compared to the rest of the plasmid (Fig. 2d). GC skew in the transfer strand—which expresses the ratio between G and C on a single strand—also often had higher variance in the leading region (Supplementary Fig. S1). Next, we investigated the possible impact of RM systems on leading region sequence composition. Because Type II RM targets tend to be short palindromes 4–8 bp in length, we initially looked at the density of short palindromes across plasmids. We compared the palindrome density expected based on GC content with the observed density (see the “Materials and methods” section). Four base-pair and 8-bp palindromes showed no clear difference in the leading region (Supplementary Figs S2 and S3). Patterns of depletion in bacterial genomes are often strongest for 6-bp palindromes [37] as also previously established for plasmids [15], and interestingly the leading region had fewer 6-bp palindromes than expected in some PTUs, such as PTU-E84, PTU-FE, PTU-BOKZ, and PTU-I1 (Fig. 3). However, many PTUs showed no signatures of depletion.

**Figure 4.**
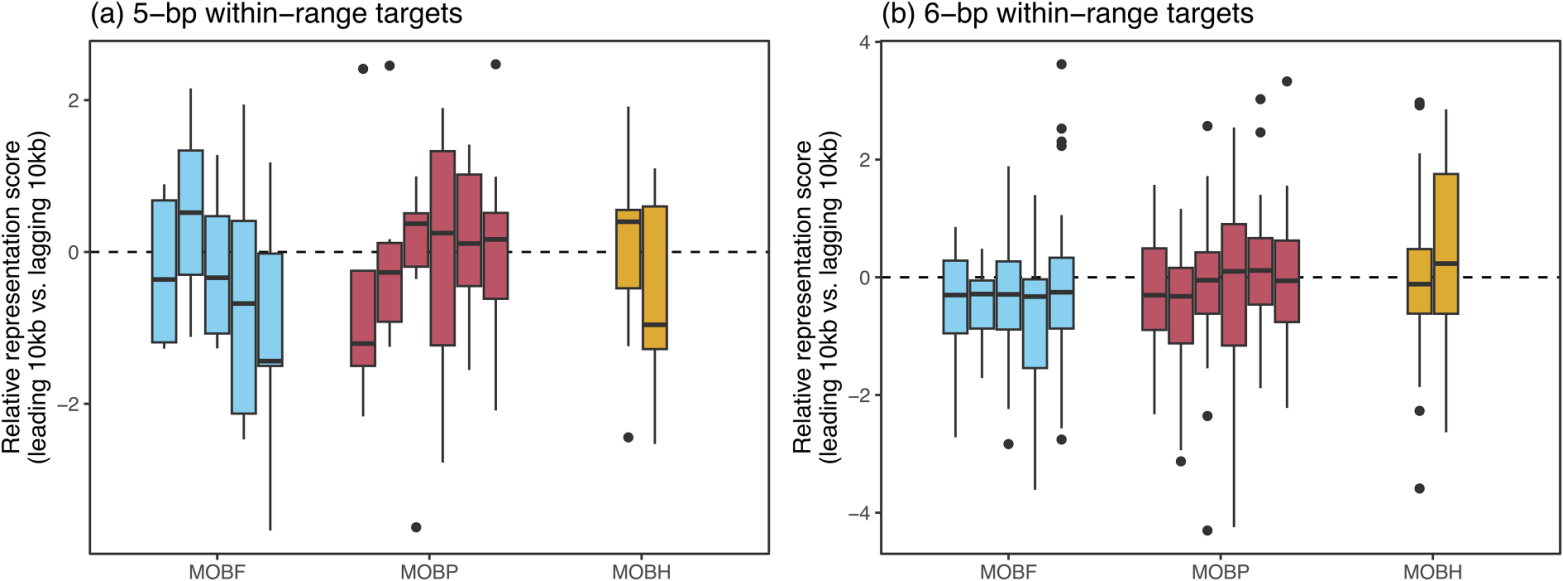
Relative representation scores for within-range RM targets in the leading versus lagging regions of plasmids. Panels show mean results for (**a**) 5-bp and (**b**) 6-bp targets of mean differences in RMES score for the within-range target between the first 10 kb (leading region) versus last 10 kb (lagging region) of all plasmids within each PTU.

Averaging over plasmids and palindromes in this way might obscure real biological signal, since different PTUs have different hosts and are therefore subject to different selective pressures. We therefore performed an analysis for each PTU for the motifs targeted by RM systems within its observed species range (‘within-range targets’), using RMES to compare the number of observed motifs to the number expected under a null model that controls for sequence composition; negative/positive RMES scores correspond under/over-representation. Since only PTU-C had a within-range target of *k* = 4, we only analysed targets of *k* = 5 and *k* = 6, comparing the leading and lagging region (Fig. 4). On average, the leading region was not depleted in within-range targets for *k* = 5 but was for *k* = 6 (scores <0 in 10/13 PTUs, one-tailed binomial test *P* = .046), in line with previous work suggesting depletion applies predominantly to 6-bp motifs ([1], [2]).

However, there is no significant mean depletion in the leading versus lagging region across all within-range RM targets and targets (Supplementary Fig. S4; mean depletion −0.14, Wilcox signed rank test *P* = .08). Furthermore, these representation scores correspond to tiny changes in number of motifs. To take a specific example, consider PTU-B/O/K/Z. These plasmids are narrow-host-range, seen only in *Escherichia* and *Shigella*. Our analysis suggested that ∼3% of *Escherichia* genomes contain Type II restriction enzymes similar to enzyme Cfr10I, which target RCCGGY = (ACCGGC, GCCGGC, GCCGGT, ACCGGT), and indeed of these four motifs, the first three are the most depleted known targets in the leading region. However, in general we found that closer examination did not reveal strong specific signals, making us cautious about over-interpreting this weak average signature. Within Enterobacterales (*n* = 17 genera), for targeted motifs of *k* = 5 (32 motifs), and *k* = 6 (89 motifs) the median motif was targeted by RM systems with only 2% prevalence (IQR 1%–4%). The most prevalent case was in *Erwinia*, where 70% of genomes had a Type II RM system targeting GTATCC, but the only plasmid in our dataset from *Erwinia* (NC_005246.1) had four instances of this motif in its leading region. We therefore conclude that there is no evidence for systematic depletion of RM targets in the leading region.

As well as potential vulnerability from Type II RM systems, we also considered the possibility that palindromes in the leading region might form transient dsDNA hairpins that could also potentially be targeted by the nuclease SbcCD [38] as suggested by Fraikin *et al*. [11]. The SbcCD complex can cleave hairpins with stems as short as 16 bp [39]. We therefore searched for any near-perfect palindromic hairpin at least 4 bp in length where the inverted repeats were separated by no more than 1 kb (einverted in EMBOSS v6.6.0.0, flags: -threshold 10 -maxrepeat 1000 -mismatch -99 -gap 12 -match 3) but found no evidence for any association of palindrome densities and position in the leading 50 kb (Supplementary Fig. S5).

### The leading region shows adapted codon usage

**Figure 5.**
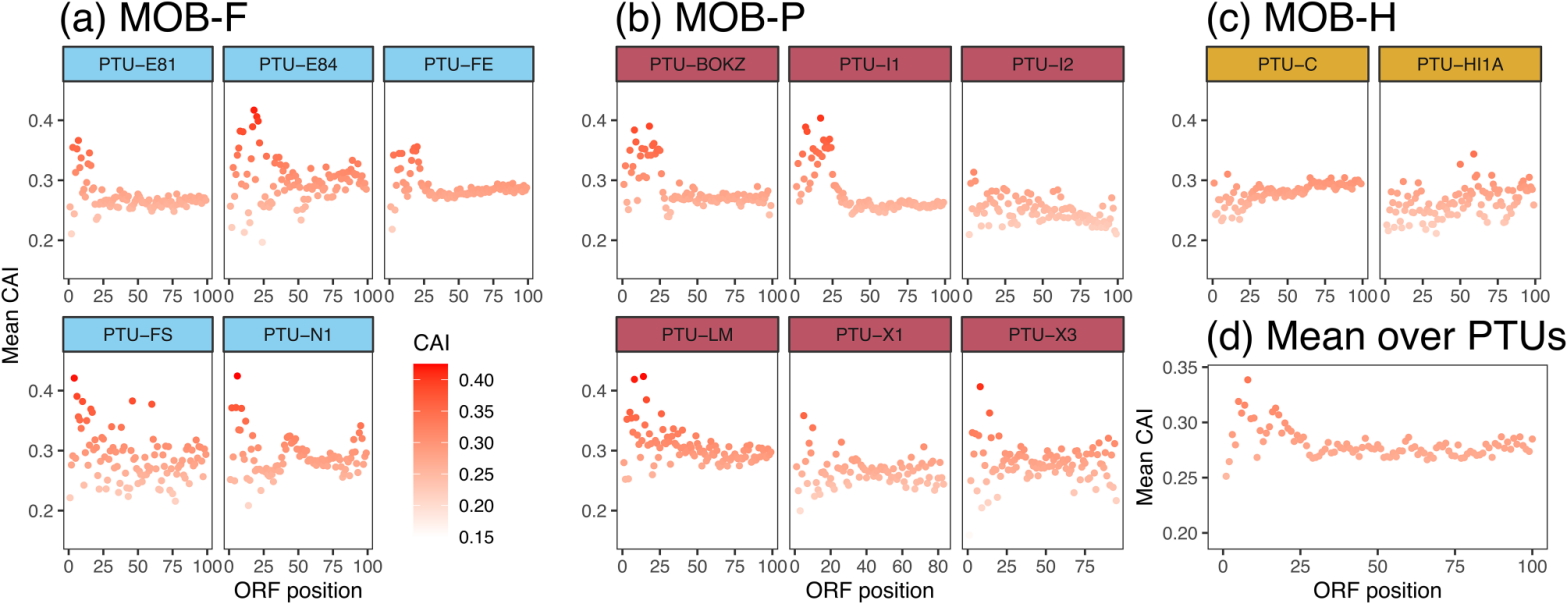
The leading region has a peak in host codon adaptation. (a–c) Plots of mean CAI by ORF position for each PTU, with *Y*-axis and colour indicating mean CAI of genes found at that position. (**d**) Mean CAI for each position averaged over PTUs (i.e. each PTU given equal weighting). Plot truncated at 100 ORFs. CAI was calculated against *E. coli* codon weights determined from highly expressed genes (see the “Materials and methods” section). PTUs are grouped by MOB relaxase type (**a**) MOB-F (**b**) MOB-P (**c**) MOB-H.

**Figure 6.**
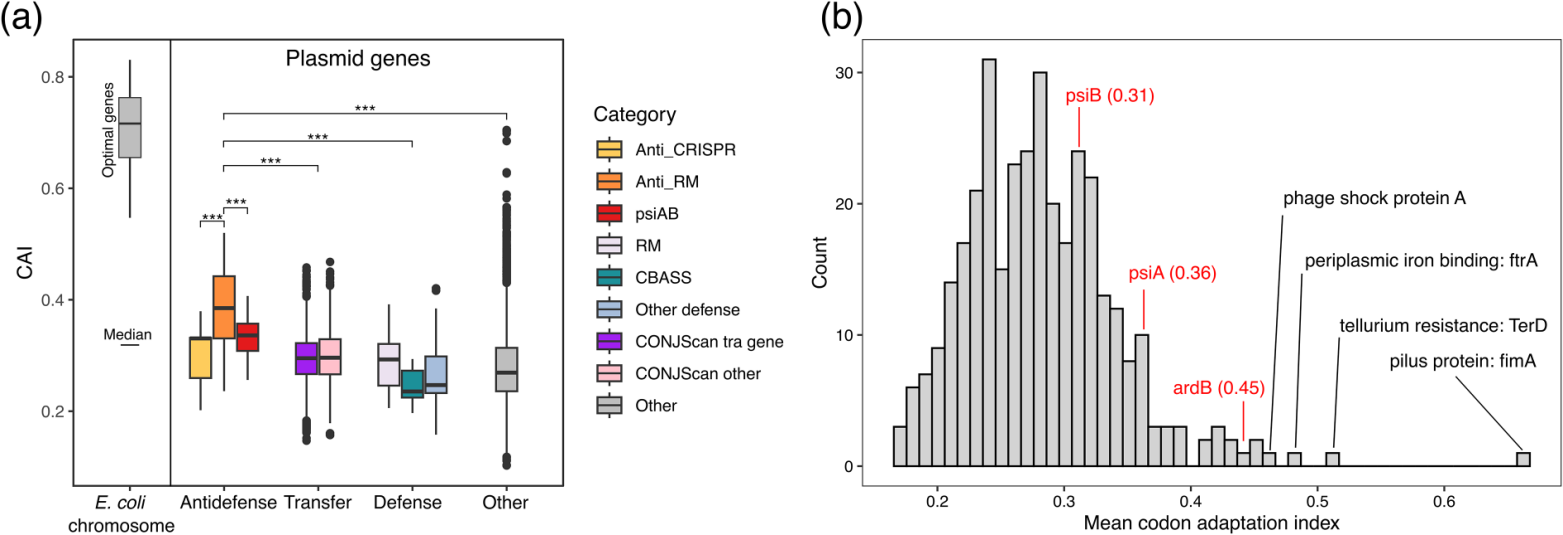
Anti-defence genes have more optimal codon usage than many other plasmid genes. (**a**) Histogram of plasmid genes from all 1751 plasmids, after classifying into defence and anti-defence (DefenseFinder), and conjugative genes (CONJScan). Data for *E. coli* chromosomal genes is shown for comparison (27 optimal set of genes used to define optimal codon weights and median). Statistical tests are Wilcoxon rank sum tests, all *P* < .001. (**b**) Histogram of mean CAI for KEGG KO categories with 10 or more gene representatives. Anti-defence genes like those encoding ArdB or PsiA/B are above average in the distribution, although there are some categories with more optimal codon usage.

For each plasmid gene, we computed the CAI relative to optimal codon usage in *E. coli* (see the “Materials and methods” section). We found a strong signal of differential sequence composition in the leading region, with higher CAI for positions in the leading region of PTUs with MOB-F and MOB-P relaxases (Fig. 5). The CAI of a gene was correlated with its GC content (Pearson’s *r *= 0.33, Supplementary Fig. S6; using GC3 produced a marginally better correlation of *r *= 0.38). This correlation with GC still held in most PTUs when only considering genes in the first 10 positions of the leading region (Supplementary Fig. S7). When we tested across plasmids, we found that their anti-defence genes had higher CAIs than their other genes (Fig. 6a; median 0.34 versus 0.27 for all other genes, Wilcoxon rank sum test *P* < .001) and were even slightly above the CAI for the average *E. coli* chromosomal gene (median 0.34 versus 0.32, *P* < .001). Within the category of anti-defence, anti-restriction genes had significantly higher CAIs than anti-CRISPR (median 0.385 versus 0.331) or *psiAB* (median 0.336, range 0.256–0.407). Analysing genes using the KEGG ontology (Fig. 6b) showed that genes with high CAIs made biological sense, including those encoding proteins for pilus formation, heavy metal resistance, iron binding, and phage shock response, with anti-defence genes having higher than average CAI. In particular, anti-restriction genes encoding proteins in the ArdB/KlcA/AcrIC11 family, which were seen across 10 PTUs in 1054 plasmids (60.7%), were highly optimized for codon usage (CAI 0.442, range 0.28–0.52). Furthermore, there was a significant negative correlation between distance from *oriT* on the leading strand and CAI for anti-defence genes (Pearson’s *r*=-0.259, *P* < .001), which was much stronger than for other genes (Pearson’s *r*=-0.054, *P* < .001).

### Codon usage patterns can help identify promoters

**Figure 7.**
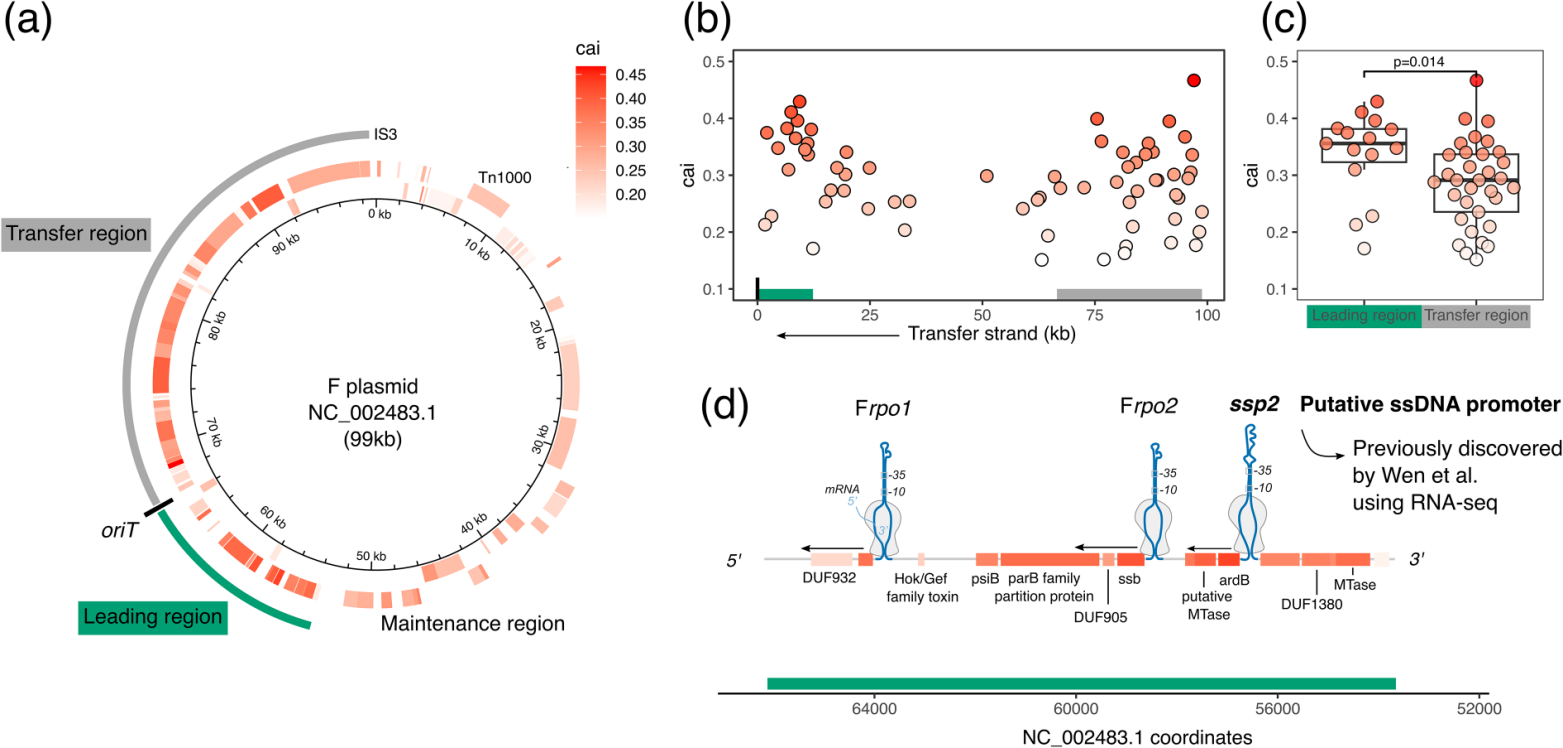
The leading region of the F plasmid is adapted for rapid expression. (**a**) F plasmid map based on NC_002483.1 using NCBI annotations (non-pseudogenes), with genes coloured by CAI. (**b**) Visualization of CAI relative to *oriT*, with sliding average of 15 ORFs shown in black (midpoint). Only genes encoded on the transfer strand are shown i.e. in the correct orientation for transcription upon entry into the recipient cell. (**c**) Leading region genes on the transfer strand have significantly higher average CAI than transfer regions (Wilcoxon signed-rank test, *P* < .05). (**d**) Single-stranded promoters in the leading region allow transcription of codon-adapted genes while still ssDNA. These include the known ssDNA promoters F*rpo1* and F*rpo2* and a putative ssDNA promoter, identified by inspecting the secondary structure upstream of the cluster of codon-adapted genes including anti-restriction gene *ardB* and a putative methyltransferase. This promoter was independently identified by Wen *et al*. using RNA-seq [44]; we follow Fraikin *et al*. in calling it *ssp2* [45]. Secondary structures for Frpo1/2 have been adapted from fig. 4 of Couturier *et al*. [10] under a Creative Commons License https://creativecommons.org/licenses/by/4.0/.

For a detailed case study, we analysed patterns of CAI in the F plasmid (Fig. 7a). Historically the leading region of the F plasmid has been defined as ∼13 kb in size [40, 41]. Genes on the transfer strand had higher CAI in the leading region (Fig. 7b), notably higher than the *tra* genes in the transfer region (Fig. 7c). The F plasmid has a known single-stranded promoter that allows transcription of genes in the leading region while they are still ssDNA. Couturier *et al*. used sequence similarity to the F*rpo* region (renamed F*rpo1*) to identify a single-stranded promoter (F*rpo2*) that shares 92% identity with F*rpo1*. This region was previously noted by Masai and Arai in their original publication identifying F*rpo* [42].

As expected, genes associated with these promoters have high CAI. However, examining the leading region in detail, we noticed that the cluster of genes including *ardB* and a putative methyltransferase had high CAI but no known promoter. Blast recovered no hits to F*rpo1* or F*rpo2*. However, when we computed the secondary structure for the DNA upstream of *ardB* on the transfer strand with mFold v3.6 [43] we were able to identify a putative single-stranded promoter. (We also searched for the region upstream of the next cluster of genes along, but failed to identify a probable promoter.) This sequence is distinct from F*rpo1* and F*rpo2* but it forms a similar secondary structure: a long hairpin within which we could identify a −10 and −35 box, making it plausible that it directs transcription along the transfer strand (Supplementary Fig. S8). We found that it also shares 86% sequence identity with a putative ssDNA promoter identified by Samuel *et al*. from an insect gut metagenome [12]. After initial publication of our paper as a preprint, we were made aware of previous experimental work by Wen *et al*. [44], which had independently identified this region as an ssDNA promoter using RNA-seq. Recent work by Fraikin *et al*. [45] has confirmed the properties of this ssDNA promoter, dubbed *ssp2*, and shown that it acts as a ‘replication roadblock’ during plasmid conversion to dsDNA: it forms a physical barrier that delays complementary strand synthesis and allows the genes under its control to be transcribed, providing compelling evidence that plasmids have evolved a precise temporal regulation of these early events during transfer.

## Discussion

The leading region of plasmids has been recently shown to be enriched in anti-defence genes. Here, we show that it is also adapted in its codon usage. Our findings are consistent with a selective pressure for the high expression of anti-defence genes during plasmid establishment, and emphasize the importance of considering how evolution has shaped plasmid genome organization. In this section we put our observations into a wider context and speculate about possible interpretations.

We considered several possible adaptations in sequence composition. First, we considered possible selective pressure from Type II RM systems, which target short palindromic motifs of dsDNA. We found that there was an average depletion of 6-bp palindromes of ∼5 per kilobase in the leading region across all plasmids, though this signal did not apply to all PTUs and was seen most strongly in plasmids like PTU-FE (MOB-F) and PTU-BOKZ and PTU-I1 (MOB-P). We then attempted to analyse the motifs targeted by RM systems that plasmids encounter most often. With the caveat that it is challenging to know how to accurately average over motifs targeted by diverse RM systems with very different prevalences and (unknown) efficiencies—or indeed what such an average really means—we found only a weak average signal of 6-bp depletion. This is consistent with previous work highlighting that depletion is largely a feature of smaller plasmids (<10 kb) since larger plasmids cannot easily lose motifs by mutation, and seem to adapt instead by carrying additional genes for evasion [15]. We therefore conclude that there is no general pattern of Type II RM motif depletion in the leading region, consistent with the fact that the leading region enters as ssDNA i.e. despite earlier exposure to host defences it experiences no differential selective pressure from Type II RM. Although it remains possible that individual instances of plasmid infection could be thwarted by certain motifs in the leading region, akin to a recent report for a dsDNA phage [46], we do not think this is a major factor in the success of plasmid transfer. We also searched for any near-perfect palindrome and found no evidence for any general association with the leading region, although it remains possible that a more fine-grained analysis of hairpins in the leading region could discover more information.

We found that the leading region of conjugative plasmids with MOB-F and MOB-P relaxases is GC-rich, with evidence of higher variance in GC skew on the transfer strand. To our knowledge these observations have not been discussed extensively in the literature, although one previous hypothesis has been made about structural requirements for DNA loading into the transfer channel using G-quadruplexes [47]. Despite unclear biological relevance, this pattern of GC content, particularly in plasmids with MOB-F relaxases, is intriguing. GC content notoriously has many possible drivers and may simply be a correlated feature of particular plasmid lineages. However, one possibility is that because GC-rich DNA forms more stable transient structures (due to the higher thermodynamic stability of GC bonds), it could have an impact on secondary structure. Since the leading region enters the recipient as ssDNA, its secondary structure during this initial phase is crucial for its function. A plasmid needs to avoid secondary structure in some stretches of sequence while having stable structures (like ssDNA promoters) where needed. In light of this, we find it interesting that single-stranded promoter regions of the F plasmid are GC-rich (mean GC content of F plasmid is 48.2% but F*rpo1/ssp1a*: 58.0%, F*rpo2/ssp1b*: 56.7%, and *ssp2*: 68.6%), whereas the start of the transfer strand typically has lower GC content. We speculate that this may reduce early transient structures that impede transfer, before higher GC in the subsequent leading region where stable secondary structures are important for the linked regulation of gene expression and complementary strand synthesis.

Considering codon usage, we found that genes in the leading region of most PTUs were more adapted for translational efficiency than the rest of the plasmid, as assessed by the CAI. GC content at the gene-level was correlated with CAI. Our interpretation of the link between codon adaptation and high expression applies best to genes with a known dose-dependent response—for example, in our dataset plasmid-borne beta-lactamases have higher CAI than other AMR genes (median 0.30 versus 0.24, Wilcox signed rank test *P* < .001). However, we stress that CAI is not a measure of a gene’s importance and that gene expression can be optimized in many other ways; in general, there is not a strong relationship between codon bias and translation efficiency because of the many other factors affecting both [20], and alternative measures of codon adaptation that account for mutation bias can also be used [48].

We did not explore the physiological dimensions of codon usage. For example, optimal codons are often ‘starvation-sensitive’: when the supply of amino acids is rate-limiting for protein synthesis, their associated tRNAs have low charged fractions and are used as control codons in transcriptional attenuation [49, 50]. This means that the optimal codons for rapid translation in resource-rich settings lead to poor synthesis under starvation when growth is throttled. Our results suggest the leading region is optimized for plasmid transfer into rapidly growing hosts, but it is possible that using rarer codons might allow better evasion under starvation conditions. This has been suggested to explain the use of rarer codons in some genes, such as in *S. enterica* where virulence genes appear to be optimized for starvation and host-mimicking conditions when compared to ribosomal genes [51]. Some plasmids and phages regulate host transcription to divert cellular resources to themselves without inducing the stringent response [52], but in general the contribution of cellular physiology to defence, anti-defence, and transfer is a complex and underexplored area. Codon usage has also been shown to affect mRNA lifetimes [53], which might be relevant when considering the continuing expression of anti-defence proteins after initial cell entry.

However, for the leading region of plasmids we argue that initial speed is the most important factor. Genes controlled by ssDNA promoters with high CAI will translate quickly, helping with the rapid conversion to dsDNA, which is important to avoid increased induction of the SOS response. Speed would be expected to be most important for anti-restriction compared to other anti-defence genes, because preventing early restriction can allow subsequent methylation to occur, protecting the plasmid from further restriction. Indeed, the high CAI of the cluster of genes containing the broad anti-restriction gene *ardB* on the F plasmid’s transfer strand led us to identify a putative promoter, which we subsequently discovered had been previously identified by independent experimental work [44, 45]. This highlights the potential value of a computational approach based on codon adaptation to identify future promoters for further experimental investigation. Future work into the relationship of initiation and elongation along the transfer strand will lead to a better understanding of how the leading region is organized in the F plasmid, and potentially beyond.

Although we calculated CAI with reference to *E. coli* as a host, fast-growing bacteria and highly-expressed genes have converged on a small subset of optimal codons and anticodons [54]. Indeed, we found codon weights for other Enterobacterales species are highly correlated (*r* > 0.9) so our analysis is not dependent on this assumption. Codon usage bias is known to be stronger for organisms with multiple habitats [55]. By analogy, the leading region of Enterobacterales PTUs may represent a sequence optimized to deal with their multiple potential hosts. Previous work on codon usage in plasmids has largely considered the effect on host fitness, such as experiments in *E. coli* that found that altering host translational efficiency did not change plasmid costs [56], or looked sat effects on plasmid retention due to codon usage mismatch [57]. By approaching the problem from the perspective of plasmid establishment, we argue that plasmid success depends not on general adaptation for translational efficiency, but specifically in the leading region.

Our results are consistent with literature that suggests that rates of horizontal gene transfer are correlated with the similarity of tRNA pools [58] and require codon usage compatibility [59]. Our dataset also had *n* = 15 PTU-C representatives found in species outside Enterobacterales and we note that codon weights for these species are not as strongly correlated with *E. coli* e.g. *Shewanella algae* (*r* = 0.814), *Vibrio cholerae* (Pearson’s *r* = 0.721), and *Photobacterium damselae* (*r* = 0.590) (based on 22 highly expressed genes in each reference genome). However, species differences seem unlikely to explain why MOB-H plasmids lack a peak in CAI in their leading region, unlike MOB-P and MOB-F plasmids, given that all tend to infect mostly the same species (Fig. 1). Other possible reasons could include different transfer dynamics or other selective pressures, but these remain to be investigated. One attractive possibility for PTU-C plasmids is that it could be related to their broad host range, with CAI perhaps relating to a tradeoff between specialization and generalism, but this reasoning does not apply to the narrow host-range PTU-HI1A.

We focused here on conjugative plasmids, but in principle a similar analysis could be conducted for the leading regions of mobilizable plasmids. However, mobilizable plasmids are ∼3× smaller [60], which could make the analysis of differential patterns across plasmid length more challenging. Likewise, a similar analysis could also be performed for integrative conjugative elements and integrative mobilizable elements, although the challenge here is accurately delimiting these elements within chromosomes.

## Conclusion

General statements about plasmids are based on two types of evidence, both of which have their own issues. On the one hand, bioinformatic analysis of many plasmids is used to make statements about the ‘average’ plasmid, often without acknowledging that this is usually just the dominant plasmid type in a (biased) dataset. On the other hand, careful experiments that characterize how one specific plasmid behaves are used to extrapolate to other plasmids, assuming that these behave similarly. Both approaches need to be weighed in the balance to understand plasmid biology. The concept of the leading region arose from experimental studies of the F plasmid but is now commonly defined bioinformatically for plasmids without experimental data. Our finding that CAI is higher in the first ∼25 kb of the transfer strand applies to the F plasmid and many other Enterobacterales plasmids, but not all: plasmids in PTU-C and PTU-HI1A (both within MOB-H) lacked any clear signal in CAI. Canonical anti-defence genes like *psiB* have been noted to be present only in narrow-range plasmids, functioning in a species-specific manner [61]. Existing knowledge of anti-defence genes is highly imperfect, but it is notable that PTU-C has very few known anti-defence systems in the leading region (most cluster at 50–100 ORFs) and PTU-HI1A had no complete anti-defence systems detected at all; this suggests alternative anti-defence mechanisms, but without knowledge of these it remains unclear how a plasmid like R27 (first isolated from *S. enterica*) is able to conjugate with high frequency into other Enterobacterales [62]. Plasmids can lack the conjugation machinery but still be mobilizable, but 94% of plasmids with MOB-H relaxases are conjugative, in stark contrast to MOB-F and MOB-P where mobilizable plasmids are significantly more prevalent and persistent [60]. This suggests that MOB-H plasmids are unsuccessful when they lack the ability to conjugate independently. Perhaps the deficit of anti-defence genes in MOB-H plasmids means that their non-conjugative versions are less able to infect new hosts through the conjugative pili of other plasmids.

Above all, our findings emphasize the need to analyse plasmids in cohesive evolutionary units such as PTUs. It is important to appreciate the differences in genome organization and evolutionary strategy between these units. The leading region of the F plasmid is GC-rich and its genes have higher CAI. These features apply to the leading regions of many—but not all—plasmids, with clear differences by relaxase type. Indeed, the leading regions of some PTUs seem to have low absolute GC, show no peak in CAI, and have a low density of anti-defence genes. This suggests that there might not be consistent features that apply to the leading region of all conjugative plasmids. More broadly, that suggests that the F plasmid might be an unreliable guide to the general dynamics of conjugative transfer. For example, although the *oriT* of PTU-C plasmids has been experimentally validated, it has also been suggested that they may carry a second *oriT*, with unknown implications [63]. The F plasmid has been the focus of recent pioneering experimental work that has demonstrated that the temporal dynamics of its leading region are complex and exquisitely controlled [10, 44, 45]. It seems plausible that other plasmids may have similarly sophisticated but potentially different adaptations; it remains important to characterize plasmids beyond the well-studied minority.

## Supplementary Material

gkag810_Supplemental_Files

## Data Availability

A github repository with analysis is available at https://github.com/liampshaw/Leading-region-adaptation. Associated data are available via Zenodo (doi: 10.5281/zenodo.20040901).
