# Supplementary material for: The leading region of many plasmids is adapted for translational efficiency
Source: Nucleic Acids Res. 2026 Aug 17;54(15):gkag810. doi: 10.1093/nar/gkag810 (PMC13478746; doi:10.1093/nar/gkag810)
Supplement: Supplementary file 1 [file gkag810_supplemental_files.zip › Supplementary-material.pdf]

The leading region of many plasmids is adapted for translational efficiency

Supplementary Tables

Table S1. Metadata for plasmids analysed in this study, including the subset of plasmids where we were confident in the location of the leading region (n=1,751).

Table S2. Detection of RM systems with known targets after screening random genomes from the 37 genera in our initial study.

Table S3. Anti-defense and defense hits detected in plasmids with DefenseFinder against HMM profiles (evalue<10^{-10}).

Table S4. Codon weights for species analysed in this study, calculated from the codon usage of highly expressed genes in reference genomes.

Table S5. Codon adaptation index for all plasmid genes (using optimal *E. coli* weights) as well as their functional annotation with eggnog-mapper.

Supplementary Figures

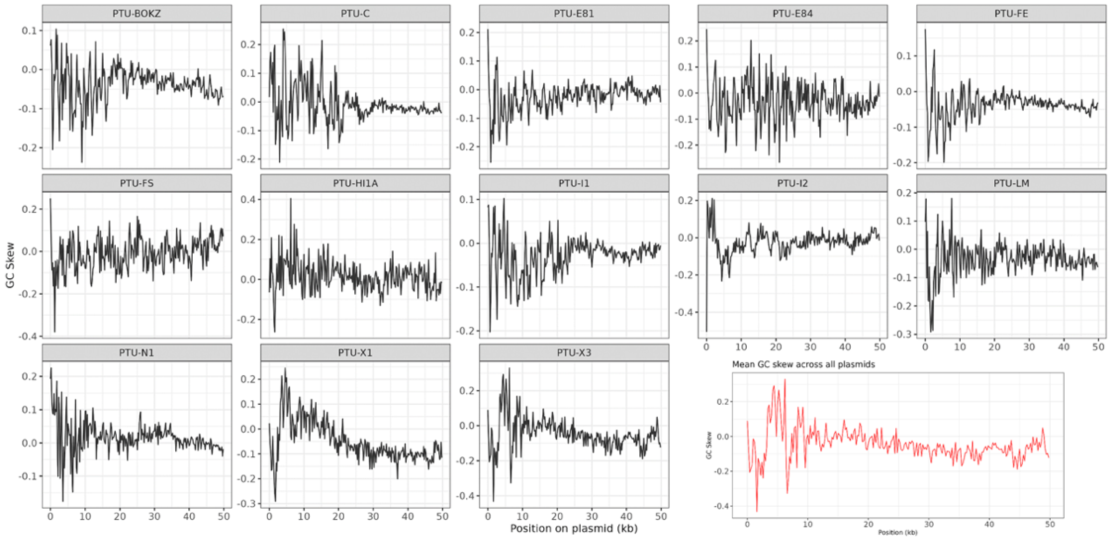

Figure S1. GC skew in the first 50kb of different PTUs. Sliding window of 200bp across 13 PTUs (1,751 plasmids), with all plasmids orientated so their leading region starts at zero.

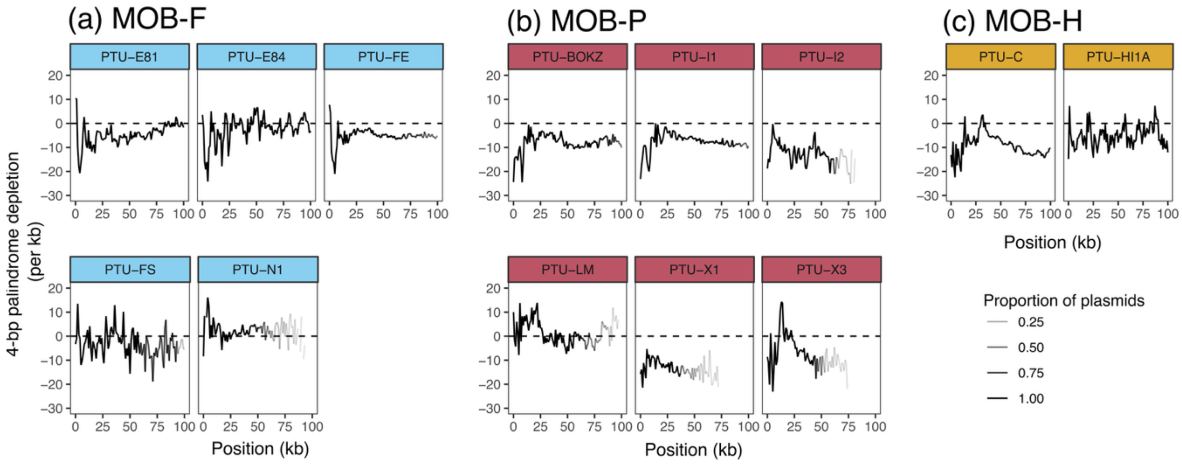

Figure S2. 4-bp palindrome density across plasmids. The difference between observed 4-bp palindrome density in nonoverlapping 1kb windows and the theoretical density expected from GC content (see Methods), such that lower values show depletion below expectation. Line intensity indicates proportion of plasmids being averaged over. PTUs are grouped by MOB relaxase type: (a) MOB-F (b) MOB-P (c) MOB-H.

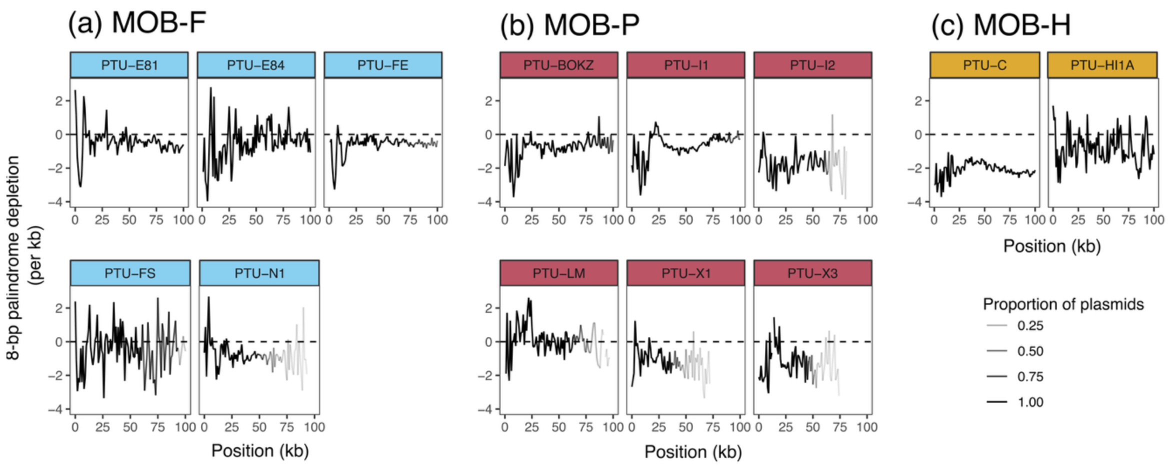

Figure S3. 8-bp palindrome density across plasmids. The difference between observed 8-bp palindrome density in nonoverlapping 1kb windows and the theoretical density expected from GC content (see Methods), such that lower values show depletion below expectation. Line intensity indicates proportion of plasmids being averaged over. PTUs are separated by MOB relaxase type: (a) MOB-F (b) MOB-P (c) MOB-H.

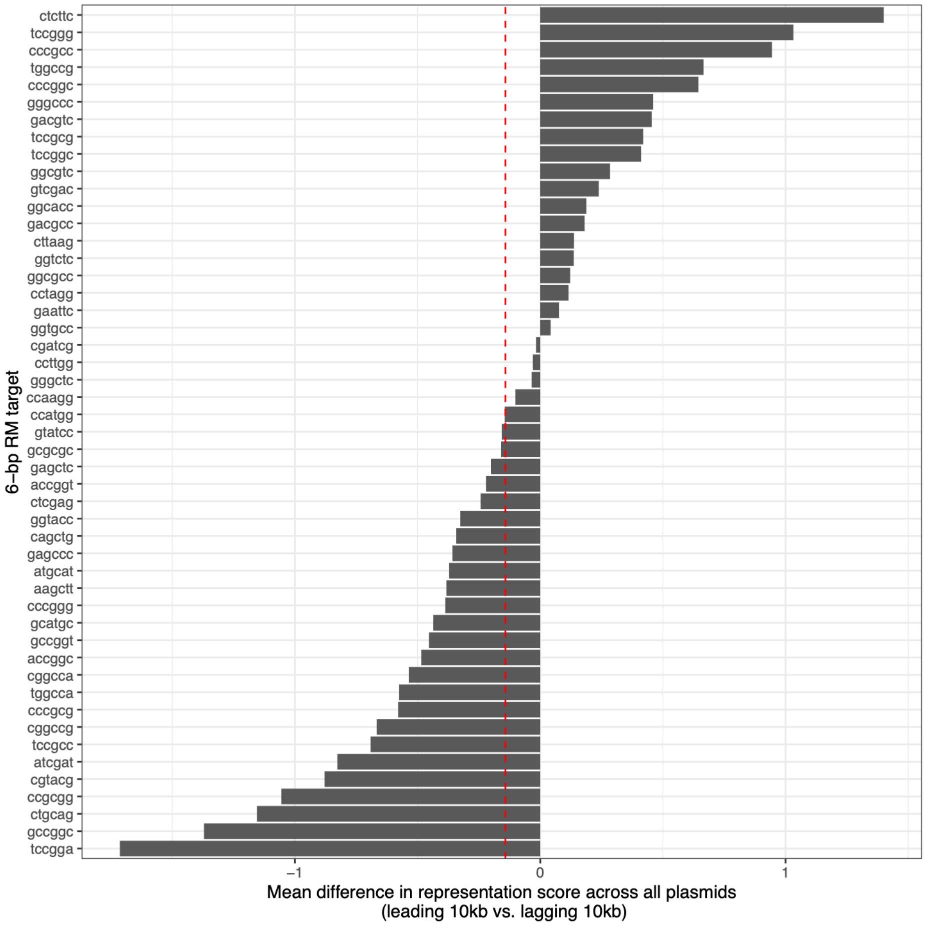

Figure S4. Depletion of 6-bp RM targets in leading vs. lagging region, averaged across all plasmids. Bars represent the mean difference in RMES score between the leading and lagging regions across all plasmids (10kb each), with negative scores corresponding to more evidence of depletion in the leading region after correcting for sequence composition. The red dashed line is the mean of the means (-0.14; Wilcox signed rank test p=0.083).

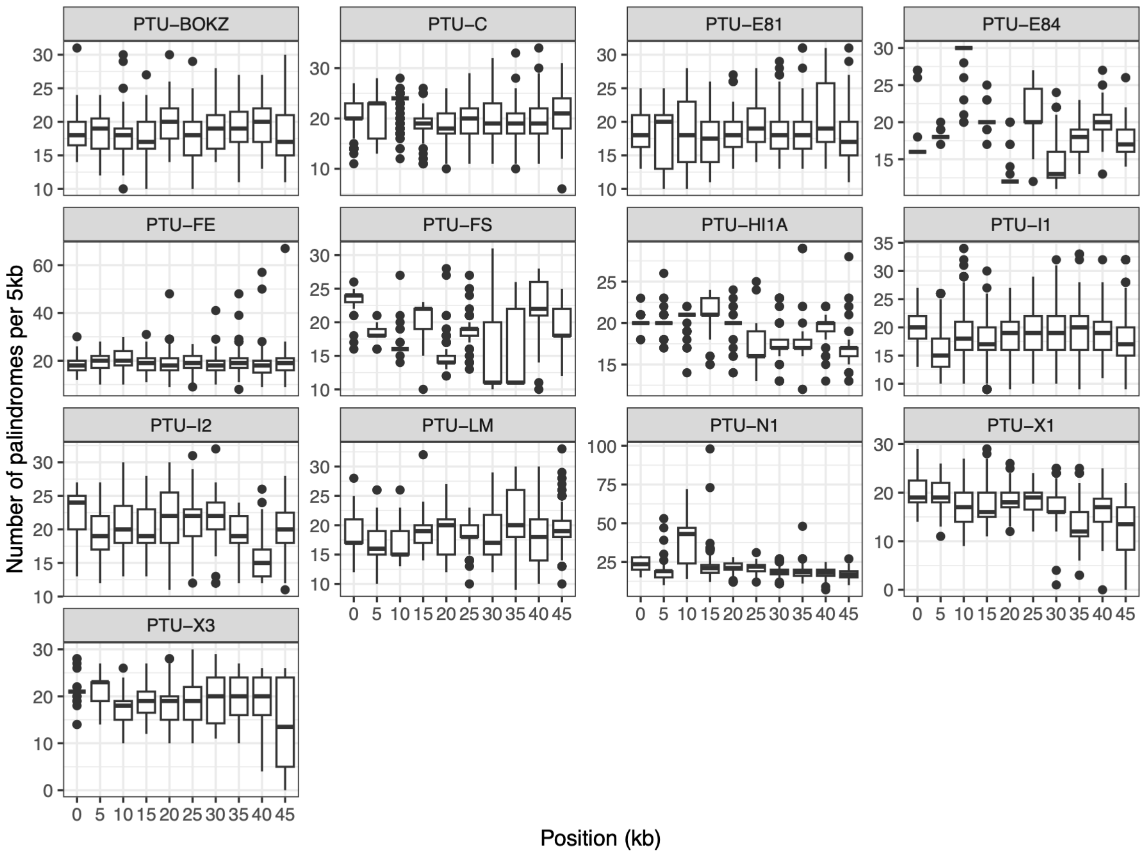

Figure S5. No variation in the number of near-perfect palindromes >4bp with plasmid position. Results are shown binned by 5kb for the first 50kb of each PTU.

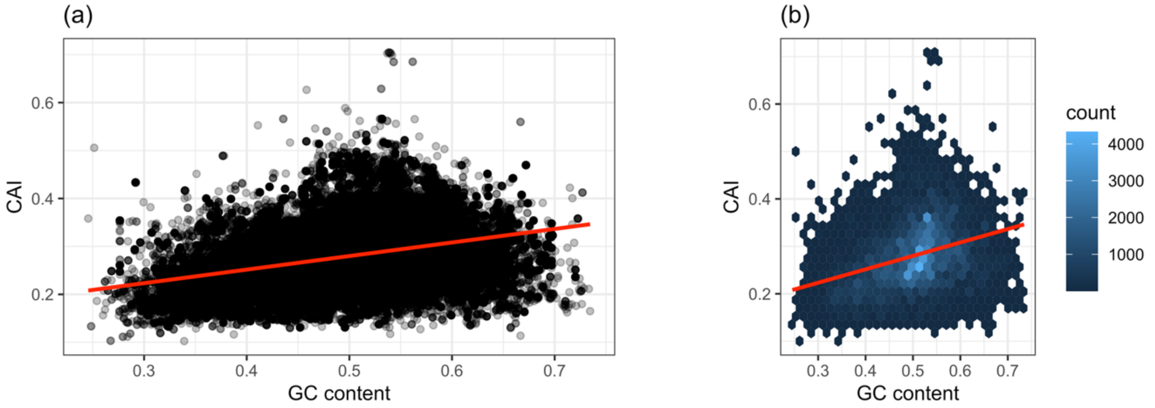

Figure S6. Codon adaptation index (CAI) is correlated with a gene's GC content. Correlation shown for 205,764 genes across 1,751 plasmids, Pearson's correlation *r*=0.33, *p*<0.001. Red line shows linear fit. Boths plots show the same dataset as (a) individual points and (b) grouped into hexagonal bins.

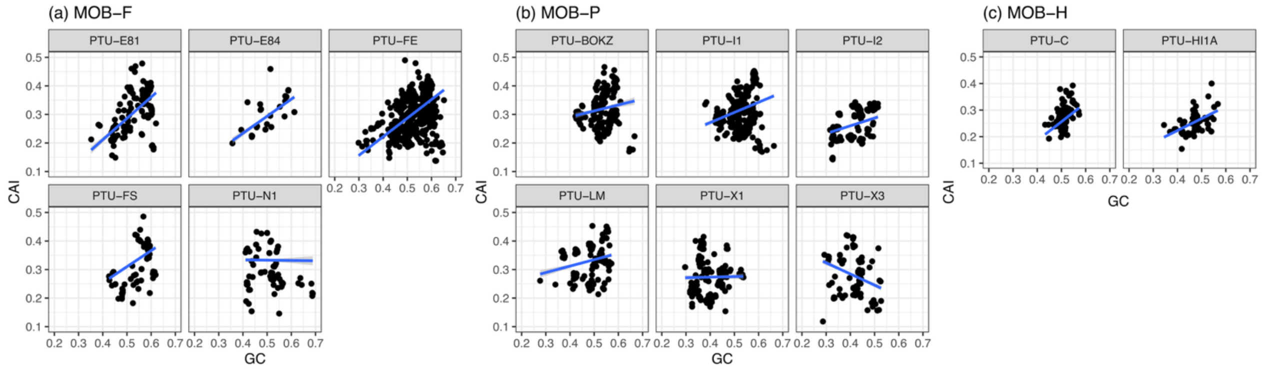

Figure S7. CAI and GC content are usually correlated for the first 10 genes in the leading region. Correlation shown for genes in the first 10 ORF positions in the leading region. Blue lines show linear fit for each PTU. PTUs are separated by MOB relaxase type: (a) MOB-F (b) MOB-P (c) MOB-H.

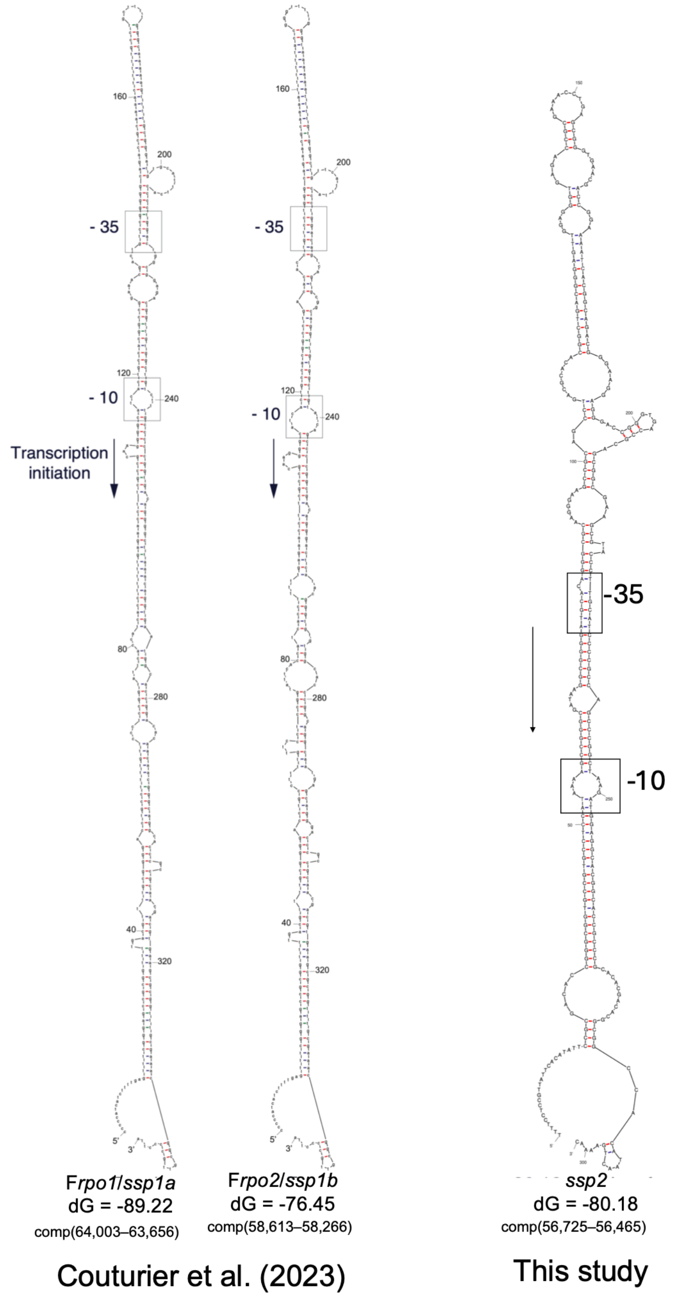

Figure S8. Secondary structures of ssDNA promoters in the leading region of the F plasmid. Secondary structures predicted with mFold v3.6 for three single-stranded DNA promoters. Coordinates are with respect to reference sequence NC_002483.1, 'comp' denotes reverse complement. The *ssp2* region was independently identified as a ssDNA promoter by Wen et al. (2025) and its function explored further by Fraikin et al. (2026), see main manuscript for full citations.
